# American Academy of Dental Science

**Published:** 1901-08

**Authors:** 

**Affiliations:** American Academy of Dental Science


### AMERICAN ACADEMY OF DENTAL SCIENCE.

The regular monthly meeting of the American Academy of Dental Science was held at Young's Hotel, Boston, Wednesday evening, February 6, 1901, at six o'clock, President V. C. Pond in the chair.

*President Pond.*—The Executive Committee has departed a little from the usual custom to-night. They have a dental subject, but presented by gentlemen of another profession; certainly a subject in which we can all be interested, as dentists, as fathers, and as public citizens. I think it is a very good idea, quite a happy thought of the Committee. Perhaps we may have granted, in some slight degree, the oft-repeated wish "to see ourselves as others see us."

I have the pleasure of presenting to you Mr. G. E. Johnson, of Andover, Mass., Superintendent of Public Schools, who will read a paper upon "The Condition of the Teeth of Children in Public Schools."

(For Mr. Johnson's paper, see page 445.)

*President Pond.*—Gentlemen, we have listened to a very interesting paper, which gives many new ideas to us, and presents other ideas, perhaps familiar, yet in a new dress. I am very glad that the Executive Committee has selected one of our ablest members to speak for us. We will be glad to hear from Dr. Brackett.

*Dr. Brackett.*—Mr. President and gentlemen, I am much afraid that on this occasion, as on others, my place may be disappointingly filled. I find, perhaps increasingly as I grow older, I have had something of the difficulty all my life, that it is hard to see all of the arguments or all of the right on one side of a question. And this matter, as it has been put before us to-night, is a thing of such breadth and of such far-reaching consequence, and it is a matter to which I have given so little thought and attention, that I do not feel able to speak as I would like to speak upon it. However, there are in my mind many comments which I would like to make.

First, let me testify most heartily my grateful appreciation of the work of the essayist, for its intelligence and good sense. If the gentleman who has presented this paper had been not only a trained man in educational affairs, as he is, but had added to that training in medicine, training in pathology, and experience as a practising dentist, you will all agree with me that he could hardly have put before us a more intelligently conceived and judiciously expressed article. Another point that is worthy of mention is the very great labor that a paper of this kind represents. He has modestly stated that it has cost time, effort, and thought in order to provide the data to give us a paper like this,—more work and more thought and more time than those of us who have not given attention to such things can fully realize.

Now, with reference to one of the points in the paper, for the sake of making a beginning,—the influence of civilization upon the physical make-up of the individual, including, with all of that physical make-up, the dental organs and their contiguous tissues. We are probably right in the generally held opinion that very many of the influences of civilization tend in the direction of physical degeneracy; and yet I do not think that idea is entirely correct, and I will give you something out of my own personal experience as a foundation for this faith which I possess.

I commenced my dental studies in the city of Holyoke, in this State, which has, and had at that time, thirty years ago this present year, when I went into the office of Dr. Taylor there as a student, a manufacturing population, partly made up of people that have not had the best opportunities in the world, not the best opportunities for physical culture, not the best homes, not the best environment, not the best diet, not the best education, not a large share of those possessions which are generally accounted among the good things of this life. They are often of a mixed parentage, French Canadians, Irish, etc., a commingling of different races, and as specimens among those races, as I have intimated, not of the best. Their material means for obtaining a generous diet were limited. Their intelligence of what constituted a diet with proper nutritious elements was limited. Their homes were not remarkable for good sanitary conditions, but rather the reverse. Working in-doors in a contaminated atmosphere, dwelling often in poor houses, with perhaps insufficient drainage, with crowding too many individuals in too small a space,—all of these circumstances together led many of them to be of small stature, of pallid complexion, and radically different from the people who come of different stock and who have lived in a different environment.

With all the rest of the physical lack, there was a most deplorable condition of the teeth. It was no uncommon experience to have children brought to the office, four, five, six, eight, or ten years of age, presenting little more than a mass of decay, hardly a tooth in the whole denture in a healthy condition, a simple aggregation of pathological expression. In some of the worst instances a large share of the deciduous teeth had lost their crowns from caries at the age of four, five, or six years, while the investments of the teeth were in such a pathological state as you can perhaps imagine. It was not an extremely rare thing among these people to find artificial dentures, nearly or quite complete sets of artificial teeth, worn in the mouths of young girls sixteen to twenty years of age. There were instances of entire upper sets being required at the age of sixteen years.

Without spending too much time upon these details, I had the opportunity to observe a very marked contrast in physical expression and in quality of the teeth in my subsequent experience in Newport. There are in Newport, among the summer colonists and among the permanent residents, a class of people diametrically opposite in their material means, in their intelligence, and in their environment from this class of people of which I have been speaking; people coming from the best ancestry, people whose mental power and physical vigor have been for generations such as have given them a foremost position among their fellows, people who have accumulated very large wealth, and who have, associated with the possession of wealth, the best and brightest minds, the best good sense, and the most intelligent capacity with reference to the rearing of their children. With this good sense and with the ample material means, there exists a condition of things that is distinctly opposite in the premises from that which I have just described; and opposite, as you would suppose, not only in the premises, but opposite in the results. The boys and the girls, the young men and the young women, that are growing up are among the very best specimens of American childhood, of American manhood and womanhood, physically speaking, morally speaking, and intellectually speaking, that are to be found in our country. It is not a very unusual thing to find in the mouths of the children of some of these families teeth absolutely perfect and mouths the picture of health during all of the period of the presence of the deciduous teeth and subsequently. I have many times seen children of these families that never had any caries of the temporary teeth, and that, as the years passed by, had only a limited amount of caries with the permanent teeth. Of course, there are marked exceptions to this rule; there are some families in which caries occurs to a very considerable extent. But, as a rule, in this case, as the rule was in the manufacturing population of lesser advantages, there is a diametrically opposite condition of things existing in consequence as there was a diametrically opposite condition of things in the premises.

We had before us an essayist, whom some of the older members will surely remember, quite a number of years ago, Dr. Stellwagen, of Philadelphia, who read a paper whose title I do not remember exactly, but it had reference to the connection between children going barefooted and the good quality of the teeth; the idea being that those children who come up barefooted, with all which that implies of open-air exercise, of rugged sports, of a good appetite and a nutritious diet, with plenty of hours of sleep, and a strong, vigorous digestion, were of the sort who would have associated with all of those things a good quality of teeth. And so, through these different links of the chain, there was a direct connection between going barefooted in childhood and a good quality of teeth in the mouth of the child.

So this is one point to which I am trying to come, that there is not necessarily associated with the accumulation of wealth, and with its refinements and luxuries, physical degeneracy. A good deal of the physical degeneracy comes in from circumstances that are not necessarily a part, and really are not a part of the best-lived life under the very highest civilization.

There are other points. As I turned the subject of this evening over in my mind during recent weeks, I questioned what was a practical thing to do in connection with this great problem which is before us, and then I questioned, what has been done in the past in the way of compulsion, or in the way of State regulation in any line, that can have comparison with that which we are discussing. And these several points come to my mind; for instance, prominently, the matter of vaccination. The State says to the child, you must be vaccinated against small-pox before you can even become a member of the public schools; and it says that wisely, and it has said it a long time, and it has said it pretty universally, and it stands to that position.

The State takes another position, and it says that if you are ill with a contagious disease of grave moment, you must be isolated so that the chances of spreading that contagious disease shall be minimized; and public sentiment supports this position.

Of course, there are instances of objection in the matter of vaccination, and there are instances of very decided objection in some localities, but it is rarely intelligent objection. Those who have been students of the history of the ravages of small-pox know that before the introduction of this safeguard against this disease, communities were decimated, and tens of thousands of lives were sacrificed needlessly, as they could not be sacrificed under conditions existing to-day. The State is justified in assuming this position.

The State makes other prescriptions. It requires that the growing generation shall be educated, that it shall not grow up in ignorance. It makes education very largely a matter of compulsion. It enacts sumptuary laws, and it says that the traffic in intoxicating liquors shall be limited or shall be prohibited, or shall be a matter of regulation in varying degrees and with various modifications in different localities at different times. It directs that the pilot of a steamship, that the engineer of a locomotive that is drawing a passenger train, or is engaged in traffic of any sort, shall be capable of distinguishing the danger signals. It says that the responsibilities of certain positions are such that only men who have demonstrated their capability shall be permitted to fill them.

In all of these things up to this point, and in some which I have not mentioned, there is to be urged the fact that these positions are taken, this legislation is had, this rigidity is maintained as a matter of self-defence. We say we will not have the whole community subjected to tendencies in a wrong direction, in a harmful and pernicious direction, from the ignorance of the rising generation, or through use, or use which can be controlled, of intoxicants. We will not have the health and the life of the community endangered by unnecessary spread of preventable disease. And in all these particulars there are the strong arguments of self-defence. The community takes all of these positions, and maintains them, as a matter of self-defence, all shutting the door against the inroad of that which may be most destructive to the mass of the people.

And then we come into a somewhat different field of legislation, in which the individual, the well-being of the individual, is considered rather more, and there is less of the principle of self-defence involved. We enact laws against the adulteration of food, against the admixture of harmful substances, or against weakening of strength, or the admixture of some substance which, if not harmful, is in the nature of a fraud. We require that various articles which are sold as food-substances and as medicinal substances shall be kept to a certain standard, or shall not be adulterated or sophisticated. In this we are not protecting the mass of the people against possible harm in just the same way that we are protecting them against possible harm when we isolate a case of small-pox; the case is somewhat different.

Then we go into another field of legislation where perhaps more of that which is rightly denominated paternalism obtains; and that is in requiring evidences of capacity of certain men in certain responsible positions who minister to others. We require that the man before he practises at the bar shall give evidence of his fitness, in a way to satisfy those who are judges of such things; and that the man who is to fill the place of a minister shall also come up to certain requirements; and in recent years we have required that practitioners of medicine and practitioners of dentistry shall be qualified for the intelligent and beneficent pursuit of their callings.

One of the foundations upon which these laws regulating the practice of medicine and the practice of dentistry rest, and a precedent which affords a strong argument for us in obtaining legislation in these fields, was the pharmacy law. Here is a case where the State steps in decidedly between the incapable practitioner of pharmacy and the individual who might possibly be harmed or is likely to be harmed in his life and health. Among the oldest of this class of laws on our statute-books, and justly so, are the laws regulating the practice of pharmacy. Filling prescriptions often involves the use of ingredients of a nature harmful, or even deadly if not judiciously compounded or if without proper directions as to quantities and intervals of repetition of dose; and the law rightly requires that this responsible work shall be done only by those whose capacity and care have been tested and certified.

In all these things the State is not only protecting itself; society is not only standing as its own protector, *en masse,* but it is doing something for the safeguarding of the individual, upon the theory that the individual is not well qualified in certain matters to be his own judge of that which is appropriate and for his advantage.

But now it seems to me that in this matter of looking out for the care of the children's teeth we should be going still farther in the direction of paternalism, and in safeguarding and helping the individual, where, in a sense, the individual interest is a large part of the interest. It is not a very dangerous thing to your family or my family that the teeth of still another family in the neighborhood are carious and are without attention. The matter is different for society, for instance, from the matter of the selling of tobacco or cigarettes to minors. There is an instance where society justly takes a position for its self-defence and for the well-being of succeeding generations. The boy who before the age of puberty, and at the age of puberty, is using tobacco is not only harming himself, but he is submitting his physical system to an influence that very probably will be manifest in succeeding generations to the disadvantage of the race. It is true that something of this obtains with reference to dental matters, as the essayist has stated, but probably to a less extent, although any legislation upon the subject of making anything in the way of the care of the teeth compulsory has to stand upon a narrower foundation than does legislation such as I have suggested.

I am indebted to the chairman of the Executive Committee, Dr. Eddy, for putting in my hands considerable literature as to that which has been accomplished in other places, and as to that which is now being done in different parts of the world bearing upon this matter. One periodical, a copy of which Dr. Eddy has placed in my hands, gives an account of the report of the committee of a Chicago dental society who sought to obtain from all of the cities of the civilized world with a population of a hundred thousand or over, statistics as to what is being done in this direction. From about half they received no reply. In more than half of the other half nothing was done. But in some few places something was done, either in the matter of lectures, or efforts to educate the people in one way or another, and in quite a number of localities efforts had been made simply to learn the condition of the teeth of the children in public schools. These efforts have been made in

Sweden, in Japan, in Belgium, in Germany, in France, and some initial steps have been taken in two or three of our Western States, Minnesota (the city of Minneapolis), Indiana, Iowa, and in Canada; but almost without exception without getting at the practical accomplishment of anything to combat the existing condition of things. The Germans, with their love for science as pure science, have limited their efforts to the collection of statistics, to learning existing conditions. A few other countries or localities have gone beyond this. Belgium, I think, in some of its larger cities employs authorized dental examiners, who perform dental operations, with the approval of the parents, for children in the public schools. But it still remains true that at the present moment, throughout the civilized world, very little is being done to combat diseases of the teeth and for the benefit of the mouths of the masses of the children.

That his condition of things does exist none of us needs any evidence to prove. A grouping of a considerable body of statistics shows, what every one of us in this room would know out of our own experience and out of our own information, that ninety-seven per cent. of the teeth of our children in the public schools are considerably diseased.

We might put with that another great fact, and that is that there probably is not ten years in the life of the individual when careful, judicious dental ministrations can accomplish more for good than in the years usually devoted to obtaining an education. I suppose that if an individual could have the best care of the teeth for only ten years of the lifetime, that ten years could be most profitably chosen in the interval from the age of six to sixteen, or eight to eighteen, or thereabouts.

Therefore, of the presence of the evil there can be no sort of question. It is an enormous thing with which we struggle, and without the advantage, without the strong arguments, without the helps that there have been in most other matters of the sort that have been ameliorated.

The chairman of the school board in Newport, Dr. C. F. Barker, an earnest and judicious man, has been for a number of years seeking to get adopted in our Newport schools a reasonable suggestion, and as yet without success, that there should be a medical inspection, at short intervals, of all the pupils of the schools upon the basis of the general welfare of the whole. There is no doubt that if the pupils in public schools were inspected by a competent physician once in two weeks, he would be able to say to this pupil, "You have symptoms of measles, or scarlet fever; you should go home and stay until this has been investigated;" or, "You have mumps;" or, "You have whooping-cough;" or, "You have parasites in your hair, or ring-worm;" or he finds some other disease which threatens not only ill for the pupil affected, but for other pupils in the schools. This suggestion, which is one of the most sensible suggestions that could be made, and for which great argument could be brought to bear, has thus far failed of adoption. I question if that idea is practised in very many of our States. Probably these gentlemen here, connected with school boards, could say whether anything of that sort is being done. Probably they will tell us later. But if there is difficulty in accomplishing so much as this, there would be difficulty in accomplishing very much in the way of compulsory care of the teeth. Another obstacle in the way is that very many families lack not only the appreciative intelligence, but they lack the means to have the work accomplished, unless it were done at the public expense; and in the present state of the public mind there would be little hope of an appropriation of public money for the care of the deciduous teeth or the permanent teeth of the children in the schools. There is perhaps a little encouragement to be found in the legislation just now accomplished after years of struggle, to provide the army with a small number of dentists.

A considerable employer of mere manual labor, that involved no great intelligence, told me some years ago that quite a share of the men in his employ, men with families, having house-rent to pay and provisions and clothing to buy, and all the necessary expenses that go to the support of a family to meet, did not earn more than two hundred dollars each in a year. They were men that were not of the ambitious sort; perhaps they did not seek to get all the work that they could get; but even with a considerable disposition to be employed, the work being out of doors, and not work to be pursued in winter when the ground was frozen, their income did not exceed two hundred dollars a year for the support of their families. Well, that cannot pay for very much dentistry.

It cannot be urged at any hearing or before any committee to investigate the subject, as an argument for the adoption of anything compulsory for the care of the teeth, that it would furnish employment for dentists. To such an argument objection would be made. The State is justified in following out this line of self-defence in a time of hardship, in a time when a good many people are out of employment, if the weather is inclement, and the times are hard, and for various reasons many people are idle, the State is justified in undertaking enterprises with the prime object of carrying them forward simply that otherwise unoccupied men may be employed, and that tendency to crime may be thus combated and repressed. The State is justified in doing that. It is not justified, except as a matter of self-defence, in giving any class of people who are not in a needy condition employment.

So that it seems to me, without continuing my remarks more than a few moments more, that a large part of what we can accomplish in the present must be here a little and there a little, in missionary effort, in cultivating a better intelligence and a better sense; and we are not without encouragement. We see progress as we watch succeeding generations of those who have come to this country. The first generation perhaps has come from a region where the advantages have been most limited, their mental caliber, their education, their material possessions, all of the most meagre sort. They have come here, and in the remaining portion of their lives have markedly improved their condition. The succeeding generation begins quite as far along, even farther along, than the old people left off, with better education, better intelligence, better appreciation of that which is helpful and advantageous for them, with more of an ambition to occupy a worthy place in the world, and their attainments are very decidedly in advance of those of their parents. In another generation or two they will take a still more advanced position, and they follow the plans and avail themselves of the advantages which a still better intelligence and more ample means enable them to command.

There must yet be some generations of school children that will not have all that we would desire them to have in the way of dental care; and it is incumbent on us to help on the good day so far as we can by such means as are justifiable when there shall be an amelioration of the deplorable condition of things which now exists.

*President Pond.*—We have other gentlemen who have certainly had a long and varied experience with school children, immense numbers of them. Whether they have devoted great thought to this special line or not I do not know, but I trust they will give us some of the results of their experience. I would like to ask Superintendent Seaver, of the Boston Public Schools, if he will say a few words.

*Superintendent Seaver.*—Mr. President and gentlemen, I was glad to receive an invitation to come here to-night, and I am very glad that I came, for I have listened to one essay and to one speech which together certainly have set me to thinking. I must despair, in the first place, of the hope that I may add anything of value to the discussion; but if what has been said has set me to thinking a little, and set me to forming the determination to do something, perhaps my being here will not be altogether in vain. I cannot help being impressed with the importance of the subject, after the able setting forth that it has had this evening.

In casting about for a few of the possibilities of doing something, it has occurred to me that, in this city, we have already in operation a system of medical inspection of the schools; and it is possible that this medical inspection might be turned in some degree towards the care of the teeth. If Dr. Durgin, Chairman of the Board of Health, who devised this system of school inspection that I speak of, were here, he might be able to say whether the twenty or thirty doctors under his direction visiting our schools daily have paid any particular attention to children's teeth. I am very sure they have paid particular attention to many things outside the list of contagious diseases. They have aided a great many children and a great many families in useful ways with reference to the care of the children, their health, their nutrition, their habits in various ways; and I should expect, if I asked him the question, that he would say that something has been done, in a general way, with regard to the care of the teeth, but I am not aware that he has collected any statistics on the subject. Certainly, in his reports to me, I do not remember that "caries" occurs in the list of diseases which the doctors have found.

I watched the operation of this system of medical inspection of the schools with a good deal of interest for several years, and I am convinced that it is doing a good and a very important work; that in a general way it is doing something to protect our children from the ravages of serious diseases and from the inconvenience of the minor troubles. There has been from the first down to, I might say, the present time a very troublesome amount of prejudice, active prejudice, and inertia, which may be called negative opposition, to overcome. The visiting physicians have been opposed in their efforts to secure proper attention to the children, from the parents and from the family physicians. This opposition shows itself in various ways. The visiting physicians, however, being for the most part skilful and tactful men, have overcome this opposition to a very encouraging degree.

I may illustrate how it happens that this opposition arises in unexpected ways. Only two or three weeks ago the principal of a school told me that the teachers in one of the primary schools in his district were very sure that it would not be worth while to bother the children and interrupt the school work by affording the visiting physician an opportunity to examine the heads of the children for the presence of small creatures which are troublesome. The teachers were quite sure that the children did not need any such examination as that. They could think of no arguments which would justify the interruption of school work that would be entailed if an examination should be allowed. But in the course of a few days three of these teachers found arguments, not *in* their heads, but *on* their heads, and they sent word to the principal of the district that, on the whole, it might be well for the doctors to examine the children's heads. In that school something over seventy per cent. of the children were found to be in trouble. They were all given a simple prescription and told they must use it before coming to school again. Thus the conservative opposition of the teachers was overcome.

One of the visiting physicians, Dr. Arnold, of Roxbury, said in one of his reports that he found marked symptoms of scarlet fever, and he told the child that he must go home and report to the parents the state of the case and ask to have the family physician called in. The child returned to school the next day, stating that the mother had declared that there was nothing the matter with the child: it might be a case of German measles, or it might be nothing at all, and that the child must be allowed to remain in school. The child was sent home again, and the visiting physician happened by accident to meet the parent, and then ensued a scene, scolding on the one side, gentle, scientific expostulation on the other, with the result that the family physician was called in, and the child was declared to be coming down with the scarlet fever.

The opposition manifested by the parents has often been serious and a good deal of trouble and inconvenience, but, as I have said, that has been more and more overcome as the years have gone on.

I can say I do not believe that the medical inspection now carried out in our schools is as thorough, systematic, and scientific as it ought to be. At the same time, I fully believe that a great deal more has been accomplished than we might have the right to expect from the expenditure made upon it by the city. These physicians are not properly compensated for their trouble, in money. The salary paid them is a mere honorarium; it is not in any sense a compensation. But these physicians are public-spirited citizens. They are willing to contribute of their time and of their skill generously to the public need, and from that spirit we are reaping a very large reward. We are getting a great deal more than we pay for.

So in this matter before us to-night, if much is accomplished, it will have to be accomplished, in the first place, by public-spirited physicians, by public-spirited scientific men, who will give their time and skill for the public benefit without reward, or, at any rate, without adequate reward, save that of the satisfaction of having done one's duty to the public in some degree well. As the essayist said to-night, the dentists in Andover have contributed of their time and skill freely, and I have no doubt that the dentists of this city, if called upon, would contribute in the same generous way.

I believe that this system of medical inspection, which we have only started in Boston, is destined to grow in the city of its first adoption, and to grow by spreading to other cities and towns, as it has already to a large degree, and that before the middle of the century we have just entered upon we shall have a thorough system of physical care of the children, which will include not only the care of the children themselves, but judicious and useful advice to the teachers; for you will find, gentlemen, that the teachers will be ready to co-operate. They will be ready and glad to receive suggestions and act upon them. What they ask for is the help that you can give. They need instruction in a general way so that they may be more ready and skilful than they are in discovering the first symptoms of the approach of disease, or of the presence of such things as deafness, abnormalities of vision, and imperfect teeth.

I remember an instance the other day which illustrates precisely what I mean. A child was reported to me as being mentally deficient and as a suitable candidate for special classes which I have for the treatment of mentally deficient children. I sent one of my experts to examine the child, and it was found that the child was normal, practically, but that it was deaf. The teacher had not discovered that fact; had not discovered that most of what was said by the teacher and by the other pupils in the room was not heard by this child. This child was, therefore, unresponsive, appeared to be ignorant, and doubtless was ignorant, but the cause was not a defective brain, but defective organs of hearing.

Another instance came to my knowledge in which a little girl had been recorded as a stupid child because, apparently, she had failed to learn what had been placed before the class day after day on the black-board. The child was so near-sighted that nothing written on the black-board could be perceived by her. The teacher had not discovered it.

Now, those teachers simply need to be told how to discover indications of that kind. The visiting physician oftentimes will, in a little talk, call the teacher's attention to the importance of looking out for these indications, and the teachers will always be willing to co-operate. It is not among the teachers so much as among the parents that the passive opposition that I have spoken of is manifested. It is true with regard to teachers, however, that they become so intensely absorbed in what they naturally regard as their main business, that they forget these things which look to them like side matters. I quite well remember my own case, as a young teacher, teaching classes in a room which was kept warm by means of an air-tight stove in which wood was burned. I used to become so interested in my lesson that I forgot to provide the stove with wood, and oftentimes my room became very cold: simply a case of absorption of my attention, which meant neglect of attention to other matters. So I suspect it is with a great many teachers. They are absorbed in what they for the time being regard as their main business, and forget these other matters relating to the health or to the physical condition of their pupils; and reminders from the medical visitors are welcomed, and they are important for the purpose of reviving attention to some other matters besides the main business.

*President Pond.*—The town of Brookline has a reputation of doing a great deal for the school children, perhaps going rather farther than many communities do in many directions. We have the Superintendent of Schools in Brookline with us, and I hope Mr. Aldrich can give us some information, something new, it may be, of something being done in this line. I have the pleasure of presenting Mr. Aldrich.

*Mr. G. I. Aldrich.*—Mr. Chairman and gentlemen, I listened with great interest to Mr. Johnson's essay. So far as I know, he is distinguished as being the only school man who has turned his attention to this particular line of thinking. As I listened I could not help wondering if he did not possess some technical knowledge in this particular matter, of which most school men are lacking. I confess that I should quite despair of succeeding as he has done in a personal attempt to learn in regard to the condition of the teeth of the school children of Brookline.

I feel as little sanguine as Mr. Seaver of contributing anything of value to the discussion; and yet it seems to me the outlook is not entirely a hopeless one. It is only in comparatively recent years that we have begun to pay very much attention to the bodies of children, to their physical well-being. Perhaps we have done rather more in the education of public sentiment than we have along the lines of positive accomplishment, and yet we have begun to surround ourselves with a good many improvements. We have educated public opinion, and in some degree this public opinion has found expression in legislation in regard to such matters as adequate floor-space for each occupant of a school-room, adequate cubic space. We are paying attention to the quantity of fresh air for each occupant of the room, and for suitable means of exhausting the foul air and supplying fresh. We are paying attention to the best means of lighting.

Then we are doing something, as has been said, along the line of school inspection. In the city of Newton, I remember, a few years ago the local board of health attempted three set medical inspections per year,—that number, simply because their funds did not allow them to undertake anything more,—but at the opening of school in September, then again after the Christmas holidays, and a short time after the Easter recess a corps of physicians went through the schools examining each pupil. The hands were examined to see if there was anything which indicated the presence of a cutaneous disease; the tongue was tested; the head was quietly examined; and I have no doubt that a medical inspection which was no more thoroughgoing, and which occurred with no greater frequency, amply paid for itself.

Along that line in the town of Brookline even more has been done, especially at times when there seemed to be a threatened epidemic of diphtheria. I am unable to say how many cultures have been made from the throats of school children, but a great many, and in a certain section of the town, a less favored section, where diphtheria seems to be almost endemic, that work has been most thoroughgoing.

But leaving those general matters, and coming a little closer to the theme of the evening, the question arises as to what may be done, and how it is to be accomplished. If I understood the essayist correctly, he hinted an opinion to which I should agree, and that is that the initiative will have to be taken by the dental profession.

As you are all aware, we have been a good deal afraid in this country of anything that savored of paternalism, and perhaps that is, after all, a rather sound feeling. I chanced to pick up a book a few days ago—my attention was attracted by the title—which was marked "Newest England," and I found it to be an account of the Australian colonies of England. To us the enterprises upon which the government has entered there, in New Zealand particularly, are very interesting and very startling. It may be urged that time has not yet elapsed to make it evident whether these enterprises are going to be successful in the long run. Certainly time has not elapsed to make it evident what the effect upon the people subjected to these various enterprises is to be; but I think we are safe in assuming—indeed, our practice during recent years makes it evident that we are getting away from that bugaboo of paternalism—that we are disposed to do more and more, to undertake in greater degree those measures which count for the welfare of the body politic.

It may be interesting to notice in this connection what can be accomplished by people who are full of zeal, even though their zeal sometimes outruns their discretion. We have in Massachusetts—have had now for a good many years—a law which runs to this effect, that instruction in physiology and hygiene, with special regard to the effect of stimulants and narcotics, shall be given to all pupils in all schools. That was brought about by the temperance workers. There is a sharp difference of opinion between this body of temperance workers and the medical body and the school people as to the wisdom of the precise methods which are attempted. I only mention the matter as indicating what can be accomplished by a body of people who are very much in earnest in regard to a given matter.

Suppose we come to this matter of the teeth. I suppose it is merely another illustration of the fact that in order to accomplish anything in a society organized as ours is, the first thing is to educate public opinion. You must impress the people with the gravity of the situation, the need of doing something. It is probably true that the average teacher knows just about as much—or just about as little—as the average superintendent of schools in regard to the general matter of the teeth of the school children. I suspect up to this time they have attracted very little attention. But teachers are an intelligent body of people, and they are very quick to respond to the need which is pointed out to them. As Mr. Seaver has said, they are, and perhaps very properly so, concerned with the main issue; but I do not know of any body of workers that is any more ready to do work in the missionary spirit, if they become convinced that by undertaking that effort they can really serve the public good.

And so, following out the suggestion of the essayist, I see no more hopeful line of approach than that one dentist, or two or three, or all the dentists in a community, should undertake to awaken and enlighten public opinion in regard to this matter. From people who lack charity, from narrow-minded people, you would no doubt hear suggestions that it was self-interest, or some other rather low motive that underlay the action, but I think we can afford to ignore criticism of that sort. And I doubt if any one of us could safely undertake to put bounds to the good which might be accomplished locally if the members of the dental profession were willing to enlist in such undertaking. It would be quite easy in any community, for instance, to get an opportunity of addressing the assembled teachers of the town or city, and that would seem to me a very suitable place in which to begin. And then quietly, indirectly, it would be quite in the power of teachers to influence a good many homes.

I have been many times surprised that in cases of children who were abnormal in the matter of sight and hearing, that fact had not been discovered by their parents. You would suppose the parents would be the first to learn of it; but those who have had to do with schools would be able to point out many instances in which the attention of the parent had been called to the fact that his child was in need of glasses, or treatment for the ear, in order that he might profit by attendance in the school. Working along that same line, I think a good deal might be accomplished in this particular direction,—viz., the care of the teeth. But evidently the land is an unknown land to us at present. It is a matter which, as I think Mr. Johnson's paper indicates, has attracted almost no attention. I confess that until I had the notice of the meeting for to-night I do not know that my own attention was ever called particularly to the matter. Somebody has pointed out three stages through which public opinion goes. He says in the first place there is the unanimity of the ignorant, then the disagreement of the inquiring, and after that the later unanimity of the wise. I take it that we are at the first stage of these three. There is at present the unanimity and the indifference which usually attends upon ignorance, the unanimity of the ignorant. The next step will be to precipitate the stage which involves the disagreement of the inquiring. We must be prepared for a good deal of opposing opinion. It will be hooted as nonsense in a good many quarters. I take it, however, that it is not at all visionary to hope for a time to come when into the purview of the medical inspector will be taken this matter of the care of the teeth. And as long as physical well-being and happiness in this world generally are so dependent upon physical conditions, it seems to me a matter which is well worth the attention of all of us.

I think, Mr. Chairman, that this is the only occasion on which I ever looked forward with pleasant anticipations to meeting a company of the dental profession. I did not know that this penalty of after-dinner talk had anything to do with the dinner. If I had, my anticipations would have been less pleasant. You are all accustomed to say, "Open the mouth, please," and so there has seemed to be nothing for us except to respond to your invitation.

*President Pond.*—A great many allusions have been made to-night to the physicians and their duties and work in this subject, and the help that they can give. We are fortunate to-night in having with us a gentleman who is a practising physician, and who has also had a good many years' experience on the school board, and perhaps he can fill a link in this discussion. It gives me great pleasure to present Dr. Green.

*Dr. Green.*—Mr. President and gentlemen, it seems to me that this association has already begun its good work. It certainly has succeeded in interesting our two friends on the left of the president, and it is to be hoped that the good work has begun through their interest in reaching teachers and parents, and ultimately the children.

It is a subject that interests me, and always has interested me; and I have been very much interested by the very carefully, elaborately prepared essay of the evening.

Now, the practical point—which I think most Yankees like to look at—is how to accomplish the work which everybody here realizes should be accomplished. I think that has been very carefully pointed out by the two superintendents of schools. They have shown us the avenues of approach, so to speak. The parent has got to be reached. The child has got to be reached by them, although probably through the teacher very largely. And then, finally, the dental profession has got to be reached, and they have got to be the ones that must reach the others, it seems to me.

Now, you know that doctors give away a large amount of their time. I am not here to sound the praises of the medical profession; but I am stating what is a well-known fact when I say that they do give away a large amount of their time, especially in cities where there are hospitals and dispensaries, to the gratuitous treatment of the sick. You know, too, that there is going out in this community a strong desire that the dental profession shall become more unified with the medical profession, that dentists shall be doctors of medicine to begin with, and that dentistry shall be their specialty, just as diseases of the skin or of the eye may be the specialties of other doctors. It is believed that dentistry should be a liberal profession; and I suppose one of the characteristics of a liberal profession is that it gives itself largely, without recompense, to the general good of the community.

Now, there is the opportunity for the dental profession, in becoming more and more a liberal profession, to do a great public work in just the direction which the essayist of the evening has shown. How can it be done? In the large cities,—take, for example, in Boston,—we have here one—I believe there are others—dental school, and in their clinical teaching they do a large amount of gratuitous work among the poor. But I imagine, although I have not definite knowledge to that effect, that children constitute a very small part of the patients. At all events, certainly in Andover, there are still many children who need the gratuitous service of a dental clinic.

Now, why should not there be in all the larger cities, in all towns, an opportunity for the children of the poor to be gratuitously treated? Why should not the dentist do as the physician does, give some of his time, even if there is not a dental school in his community, towards taking charge of these children of the poor?

That is one way in which the dental profession can become more and more a liberal profession. When such a clinic is formed, why should not it be found that these clinics would attract dental students who would come to this little coterie or gathering or society of dentists who have come together to form a free dispensary? Why should not dental students come to them for their dental teaching? It seems to me that there is an opening for dentists to become professors of dentistry, although not connected with a large dental school.

Then again, dentists can reach the parents, as the essayist of the evening has said, through talking to mothers' clubs. I am very sure that a great deal of the time of women's clubs must be spent much less profitably than in listening to the instruction of the dentist in pointing out this condition of affairs in regard to the care of children's teeth. If the parents can be thus interested, their objections can be removed.

Then, again, the teachers can be reached. There are teachers' meetings in all the counties, if not in the towns, certainly once or twice a year. Why should not a dentist be chosen among the fellows of his society in the different towns to speak to these teachers, and to say to them just what the essayist of the evening, Mr. Johnson, has said to us to-night, and inform them?

Now, a teacher may not be able carefully to examine the children's teeth; but the teacher can surely tell whether the children's teeth are clean. The teacher can find out whether that child has ever been taught to brush his teeth, and how often; whether, as one child said, he brushes his teeth only on Wednesday and Saturday afternoons and Sunday morning. I remember a teacher once in my hearing saying to a boy, "My boy, your teeth are not clean,"

and the boy looked ashamed. I have no doubt he attended to his teeth better after that. Teachers could be reached through these teachers' meetings, as we have been reached here this evening; the teachers could do a great deal without the aid of the medical inspector, and after they are interested they would be willing to help in this matter. Probably not half of the teachers have ever thought of this subject.

I do not know, Mr. President, that I can add anything to what I have said, but I do feel very much gratified to think that this subject has been considered here this evening. I do not know that it has ever been spoken of before. I have never heard it so fully spoken of before. And yet it is a subject I have long been interested in, as in all other things that affect the poorer classes. It seems to me that it must be a stimulation to every member of the dental profession here to-night to think what the possibilities are of doing a very great good in his community in taking the initiative in overcoming the obstacles in reaching the teacher, the parent, and ultimately the child, on this important matter.

*President Potter.*—The subject is open for discussion.

*Dr. Potter.*—I have been very much interested in the course of this discussion, especially in the closing remarks of Dr. Green. I thoroughly agree with him that the dental profession must liberalize itself by a free service to those who need it. It is very well for us to go before teachers and tell them that all poor children need the best of care for their teeth. What is the use of making this statement, unless we are able to meet their needs?

Their needs can be met in some places, perhaps, by the local dentists organizing themselves and giving a part of their time every week for the free treatment of such cases. In the large cities the dental schools can take a considerable share of this sort of work. We who are connected with either of the dental schools in this city know that many children are there taken care of; their teeth, which would otherwise go to ruin, are filled. If we can arouse teachers and parents to send poor children for dental treatment to places which are now in some places provided, we will accomplish much.

*Dr. Ainsworth.*—Two or three thoughts come to me in connection with this matter. In the first place, I wish to compliment the essayist on the ability he has shown in writing this paper. It seems astonishing that a man who has made no special study of dentistry could put on paper the essay that he has given us to-night.

The next thought is that our friends outside the profession are perhaps inclined to grasp this subject a little too high up, not getting at the root of the trouble. It seems to me that we can reach this matter better through the gospel of cleanliness. It is an accepted fact, I think, that if we could establish perfect cleanliness, we should have very little decay of the teeth,—that is, teeth of the better quality. If we can instruct in the schools on these lines the importance of cleanliness of the mouth, we would save the necessity of much of the work and expense that comes later.

It is probably a fact that the mass of the poorer people omit having dental work done on account of the expense. Perhaps some think it is economy to omit the expense of a tooth-brush; if so, I think we might supply tooth-brushes to the children. But, at least, if we could reach the teachers, and impress upon them the importance of cleanliness of the mouth, that they may inculcate it in the minds of the pupils and awaken in them a little pride about their personal appearance, we should start the ball in the right direction.

There are very few people who understand that the loss of a tooth is anything more than the loss of a tooth. The sixth-year molars are the buttresses to the arch, and stand as a support to the rest of the teeth in the arch. Each tooth is necessary to the arch, more particularly, perhaps, the cuspid teeth, which come in when the others are in proper condition and key up the arch. Now, the loss of any one of these teeth results in a sagging in of the whole arch. Then that arch does not properly fit the other arch, may it be upper or lower, and in consequence it cannot perform the work that it is designed to perform to the same advantage. No one would think of competing, in a delicate piece of machinery, a pair of cogs with one tooth broken out against a pair where every cog was perfect. He might run the machine for an indefinite length of time, but it would not run with the same quiet ease that the one with the perfect cogs would. It would shake itself to pieces; it would shake the building down in time. And so with the teeth; if you lose one tooth from the arch, you have put that arch in a way to shake itself to pieces, and with it the whole system.

Then it seems if we could start in by teaching the gospel of cleanliness to the children, we should start in the right place.

*Dr. Hulme.*—In making that examination of the children in Andover, it was interesting to notice how interested the children themselves were in the examination. They, almost without exception, came to be examined practically without fear. As you know, children have a great dread of the dentist, but they came to us practically without fear, and were examined pretty thoroughly. They went home and told their parents of their condition. In some cases diagrams of their mouths were given them. They were taught what the marks on the diagram meant: cavities were marked in certain ways, good teeth were without marks, and extracted teeth marked in a different way, and so on. They were told when their condition was good, when fairly good, and when bad. They went home and told their parents. The parents were, of course, more or less interested, and in some cases it did a great deal of good; in others they let it drop as a thing to be put off until to-morrow.

If these examinations could have been carried on several times in the course of the year, I think they would have accomplished a great deal of good; simply the examination, and the instruction given to the children and carried home to the parents. You can interest the parents only by interesting the children, and not in the other way, by reaching the parents first and the children afterwards. You must reach the children first, and if the children's teeth can be saved through their own interest in them, the parents are perfectly willing to help all they can. Of course, they had a dread of the filling of their teeth,—that has been drilled into them from time immemorial,—but after a short time spent in the presence of the teacher and the superintendent they got over that dread enough to come, for the examination at least, without any great reluctance.

*Dr. Smith.*—I think one point is overlooked, in discussing the question,—namely, that gratuitous service to the poor on the part of the dentist means much more in a money way than gratuitous service on the part of the physician. The physician in making his calls upon the sick can, without much pecuniary loss, drop in upon a poor patient here and there. An hour of his time given in this way would enable him to see several patients. With a dentist it means much more: an hour of his time would hardly suffice for one patient. If the dentists in the town of Andover should undertake to properly care for the teeth of the poor children in that town, they would have to relinquish their private practice.

As has been pointed out by other speakers here this evening, the two dental schools in Boston offer an opportunity for poor children to be treated free of charge. If some scheme could be put into effect by which the children would be obliged to show to their teacher a certificate from one of these schools stating that their mouths had been properly inspected and cared for, it would seem to me a step in advance. In my own clinic in the Harvard School we find too commonly, as the essayist has pointed out, that the first permanent molars are gone beyond any chance of saving, and the loss of these teeth is many times a serious matter in the treatment of irregularities of the teeth, entailing upon these patients very much more regulating than would be necessary were these first permanent molars kept in a good condition. As the essayist has well said, if we could only bring it about so that these children could be brought to these infirmaries while young, before losing their temporary teeth, and before the eruption of the first permanent molar, it would add much to their health, and avoid trouble

*Dr. Green.*—Mr. President, I wish to add a word in reply to Dr. Smith. I am afraid he does not realize altogether what he says, when he says that the dentist would be giving more than the medical profession. I do not suppose that the public generally, and perhaps not the dental profession, realize what the men who work in hospitals do give to the poor. It is not, as Professor Smith has intimated, that the physician, making his round of visits, with his carriage, can stop now and then and make a call upon a charity patient. It is true, it would not take as much time as it would take a dentist to attend to one tooth in a poor child's mouth. But how about the surgeon, who spends the entire forenoon at the hospital? There are a good many surgeons who are on duty four months, certainly, in the year, and during that four months that they are on duty they spend practically the entire forenoon of every day, Sunday included. That is a pretty good part of the working year.

It is surely not a matter of a minute now and then, or ten minutes now and then, with surgeons. It means a very large expenditure of time. Now, as regards the physician who attends the poor in the hospital or dispensary, he spends a good part of his forenoon in visiting in the medical wards of the hospitals. And that is being done in Boston right along by physicians and surgeons who spend, not ten minutes now and then, but an entire forenoon during three or four months of the year.

In any community it would be a little hard for one dentist to give all the time necessary for the care of the teeth of all the poor children. I suppose most of the children are poor. We think here in Boston all the sick people are poor. But if a number of dentists gave each a little time, large numbers of poor children could receive appropriate attention.

*Dr. Smith.*—I would not in my remarks be understood to underestimate the value of the gratuitous services of the physicians or the surgeons, but if we are to praise the gratuitous work of the physicians and surgeons in Boston, or any large city, we should, with justice, speak of the gratuitous work on the part of the dentists. We have dentists who are giving a great deal of their productive time to the dental infirmaries. We have demonstrators in the Harvard School who give the entire forenoon every day in the week. We have Dr. Eddy here from Providence, who gives an entire day once a week, which is practically a free service; the salary which he receives amounts to almost nothing.

It is also true, I think, that the young dentist becomes a busier man in his practice earlier than the young physician or the young surgeon, and therefore the young physician and the young surgeon can afford to give their time better than the young dentist. And then again, the young surgeon needs his hospital experience, and on that account is always very glad of the opportunity to give all the service he can.

*Dr. Brackett.*—There is this difference between the practice of the dentist and the practice of the physician: the physician visits the patient, investigates the case, and directs what shall be done. The dentist sees the patient, and has to go to work and do with his own hands what has to be accomplished. There is no nurse or relative or attendant who can carry out the dentist's directions. He must take his own time, and he must do with his own hands that which is to be done. There are instances, of course, where the physician performs the functions of the nurse, but, as a rule, there is this difference between the two practices: the physician is a man who directs what others shall accomplish; the dentist has to go about the labor himself.

*President Pond.*—There is another point, and that is the amount of work. I hardly think any physician will claim there are ninety-seven per cent. of the children needing his services. There are ninety-seven per cent. of the children needing our services. With such an immense amount of work to be done, and so few to divide it among, you will have to have some different scheme from anything that has yet been proposed, it seems to me.

Is there anything further?

*Dr. Clapp.*—I should like to move a vote of thanks to the gentlemen who have so kindly favored us this evening, and given so much instruction.

Voted.

On motion, adjourned.

CHARLES H. TAFT,
*Editor American Academy of Dental Science.*

